# Genome Sequence of Salmonella enterica Serovar Typhimurium Phage SAP12

**DOI:** 10.1128/mra.01086-21

**Published:** 2022-05-09

**Authors:** Khashayar Shahin, Abbas Soleimani-Delfan, Lili Zhang, Abolghasem Hedayatkhah, Mojtaba Mansoorianfar, Hongduo Bao, Mohadeseh Barazandeh, Jalil Enteshari, Ran Wang

**Affiliations:** a Institute of Food Safety and Nutrition, Key Lab of Food Quality and Safety of Jiangsu Province-State Key Laboratory Breeding Base, Jiangsu Academy of Agricultural Sciences, Nanjing, China; b Key Laboratory of Phage Research, International Phage Research Center, Jiangsu Academy of Agricultural Sciences, Nanjing, China; c Curtin Medical School, Curtin University, Perth, Western Australia, Australia; d Suzhou Institute of Nano-Tech and Nano-Bionics, Chinese Academy of Sciences, Suzhou, China; e Department of Cell and Molecular Biology and Microbiology, Faculty of Biological Sciences and Technology, University of Isfahan, Isfahan, Iran; f Jiangsu University, Zhenjiang, Jiangsu, China; Portland State University

## Abstract

Here, we report the genome of phage SAP012, which was isolated against Salmonella enterica serovar Typhimurium. The SAP012 genome is 59,618 bp, with a G+C content of 56.2% and with no antibiotic resistance or virulence genes, and is quite similar at the nucleotide level to a number of previously sequenced Salmonella phage genomes, e.g., GenBank accession numbers KM366098.1 and KC139515.1.

## ANNOUNCEMENT

Salmonella enterica serovar Typhimurium is an invasive pathogen that causes salmonellosis in poultry and humans. Antibiotic therapy is the first line of prevention, control, and treatment measures against salmonellosis. Due to the cost of antibiotic therapy and the antibiotic resistance crisis, finding cost-effective alternatives is vital (1–4). SAP012 is a bacteriophage that was isolated by adding raw sewage (Isfahan, Iran) to the early exponential phase of *S.* Typhimurium ATCC 14028 at 37°C for 24 h, with shaking at 150 rpm, and then was filtered through 0.22-μm syringe filters (JinTeng, China). The lytic phage was selected based on the presence of clear plaques on *S.* Typhimurium ATCC 14028, and a single plaque was purified using three repeats of the double-layer agar method as described previously (5, 6). The genomic material was extracted following the method described by Soleimani-Delfan et al. (7). The phage DNA library was prepared with the Nextera XT library preparation kit (Illumina, San Diego, CA) and sequenced on the Illumina HiSeq 2500 platform with 150-bp paired-end reads (TGS Co., Shenzhen, China). The reads obtained (8,142,574 total reads, consisting of 1,221,386,100 bases) were imported to CLC Genomics Workbench v12 (CLC Bio, Aarhus, Denmark), quality control and adapter trimming were conducted with a quality control and read trimming pipeline with default parameters, and low-quality reads were removed. The high-quality trimmed data were assembled with a *de novo* strategy using CLC Genomics Workbench v12 with default settings and were scanned against the NCBI nonredundant database using BLASTn to characterize the taxonomic relationships of the phage (8). Open reading frames (ORFs) were identified by GeneMarkS (9) and scanned against the NCBI nonredundant database using BLASTp (8). The genome was annotated based on PHASTER, BLASTp, and ExPASy protein BLAST results (10–12). The tRNA sequences were determined by tRNAscan-SE (13). PHIRE was used to identify the phage promoters (14), and the Rho-independent factor was highlighted using the ARAGORN heuristic detection algorithm (15). Virulence factors were detected by ResFinder v4.0 (16), and antibiotic resistance factors were detected by using the Antibiotic Resistance Genes Database (ARDB) (Center for Bioinformatics and Computational Biology, University of Maryland) (17).

The Salmonella phage SAP012 genome is a 59,618-bp linear double-stranded DNA (dsDNA) genome with a G+C content of 56.2%, 931,200 total reads, and 23,360× average read coverage. The genome includes 72 ORFs, with limited similarity at the nucleotide level to Salmonella phage FSL SP-030 (GenBank accession number KC139519.1) (76.85% identity and 30% coverage) and Salmonella phage FSL SP-039 (GenBank accession number KC139514.1) (76.85% identity and 30% coverage). No tRNA sequence was detected for SAP012. The SAP012 DNA polymerase and terminase large subunits are similar at the amino acid level to those of *Providencia* phage Redjac, with 80% (GenBank accession number YP_006906013.1) and 83% (GenBank accession number YP_006906015) identity, respectively. The major capsid protein showed 82.37% identity and 100% coverage with respect to Salmonella phage Chi (GenBank accession number YP_008058174) (18).

### Data availability.

The Salmonella phage SAP012 genome is available in GenBank under the accession number NC_053008.1, with the NCBI SRA accession number SRP333849 (BioProject accession number PRJNA756012).
